# Influence of Extremophiles on the Generation of Acid Mine Drainage at the Abandoned Pan de Azúcar Mine (Argentina)

**DOI:** 10.3390/microorganisms9020281

**Published:** 2021-01-29

**Authors:** Josefina Plaza-Cazón, Leonardo Benítez, Jésica Murray, Pablo Kirschbaum, Edgardo Donati

**Affiliations:** 1 Centro de Investigación y Desarrollo en Fermentaciones Industriales, CINDEFI (CCT La Plata-CONICET, UNLP), Facultad de Ciencias Exactas, 50 y 115, La Plata 1900, Argentina; joplaca@hotmail.com (J.P.-C.); leonardo_benitez26@yahoo.com.ar (L.B.); 2 Instituto de Bio y Geociencias del NOA (IBIGEO), CONICET-UNSa Av. 9 de Julio 14, Rosario de Lerma 4405, Argentina; murray.jesica@gmail.com; 3 Cátedra de Suelos, Carrera de Geología, Facultad de Ciencias Naturales, Universidad Nacional de Salta, Av. Bolivia N° 5150, Salta 4400, Argentina; pkirschbaum@gmail.com

**Keywords:** acid mine drainage, biooxidation studies, Pan de Azúcar mine

## Abstract

The risk of generation of acid drainages in the tailings of the Pan de Azúcar mine that closed its activities more than three decades ago, was evaluated through biooxidation studies using iron- and sulfur-oxidizing extremophilic leaching consortia. Most of tailings showed a high potential for generating acid drainage, in agreement with the results from net acid generation (NAG) assays. In addition, molecular analysis of the microbial consortia obtained by enrichment of the samples, demonstrated that native leaching microorganisms are ubiquitous in the area and they seemed to be more efficient in the biooxidation of the tailings than the collection microorganisms. The acid drainages detected at the site and those formed by oxidation of the tailings, produced a significant ecotoxicological effect demonstrated by a bioassay. These drainages, even at high dilutions, could seriously affect a nearby Ramsar site (Laguna de Pozuelos) that is connected to the Pan de Azúcar mine through a hydrological route (Cincel River).

## 1. Introduction

Acid mine drainage (AMD) is a highly acidic wastewater rich in heavy metals and metalloids. Its generation at abandoned and operating mining sites is a serious environmental problem since it can affect the surrounding ecosystems, human infrastructure, and freshwater [1,2,3]. AMD is the result of the oxidation of some sulfide minerals (mainly pyrite) when they are exposed to oxygen and water [4,5,6].

The first step in the formation of AMD is the oxidation of metal sulfides such as pyrite that are capable of generating acidity. This reaction involves oxygen as the first oxidizing agent. The ferrous ion, one of the products of that process, can be slowly oxidized to ferric ion under abiotic and acidic conditions. The presence of iron-oxidizing microorganisms can dramatically accelerate this oxidation process. These extremophilic microorganisms can get energy from such reaction even at pH below 1–1.5. Bacterial species of the genera *Acidithiobacillus* and *Leptospirillum* are the most extensively studied within these extremophiles because they are ubiquitous in those environments [7]. Within the domain of Archaea, *Ferroplasma* spp. have been the most frequently detected [8,9,10,11,12]. However, the acidophilic communities involved in AMD generation are very complex and include several microbial species; their composition and dynamics under different conditions have been widely documented [13,14,15,16,17,18,19,20]. In any case, the main role of microorganisms is the continuous supply of ferric iron, which is a powerful oxidizing agent able to replace oxygen and to cause a significant increase in the pyrite oxidation rate [21,22]. The ferric attack takes place through a series of steps in which the first and main intermediate is thiosulfate which can also be oxidized to sulfuric acid by sulfur-oxidizing microorganisms. Other metallic sulfides can also be oxidized through similar processes by releasing the respective metals into solution. In some of those cases, polysulfides and elemental sulfur can be the intermediates that are also oxidized by the action of the same microorganisms [23,24]. Finally, ferric ion can precipitate as different solid phases increasing the acidity of the medium. Jarosite (MFe_3_(SO_4_)_2_(OH)_6_) is the dominant phase when the pH is below 2 [20].

Due to the low profit margin, several metalliferous mines located in the north of Argentina have limited their activities in recent decades and many of those mines were inadequately closed. Pan de Azúcar Mine, located in the northwest of Argentina (Jujuy province), at 3700 m above sea level (22°32–22°38 S and 66°01–66°08 W) is one of them. The extractive activities for lead and silver recovery finished in 1990 [25]. The three tailings dams (DC1, DC2, and DC3) were built near the old tailings accumulated at the site. Currently, DC1 does not present signs of erosion. On the contrary, DC2 suffered strong erosion that caused it to break in different places and flow downstream covering DC3. During the wet season, water drains through the tailings forming an acid lagoon downstream. The Pan de Azúcar mine is very close to Laguna Pozuelos (National Natural Monument, Biosphere Reserve and Ramsar site) whose neutral to alkaline water hosts 44 species of birds. The Cincel River, the main tributary of the lake, connects both sites since it crosses the mining area; a little change in its composition could have a dramatic effect on the local flora and fauna of the area [26,27].

Precisely, the assessment and characterization of acid drainages associated with the tailings in Pan de Azúcar mining area were carried out in this work, which included a bioassay using *Lactuca sativa* for monitoring the ecotoxicological risk. Besides, the risks of acid drainage generation in the different zones of the stratified tailings dam (DC2) were analyzed through a biooxidation test using extremophilic acidophilic microorganisms. Its results were compared with those of the NAG test, which is the usual static test to predict the generation of acid drainage. Finally, the ability of native and collection microorganisms to oxidize the tailings and the role of iron- or sulfur-oxidizing microorganisms were compared.

## 2. Materials and Methods

### 2.1. Description of the Site

**Figure 1:**
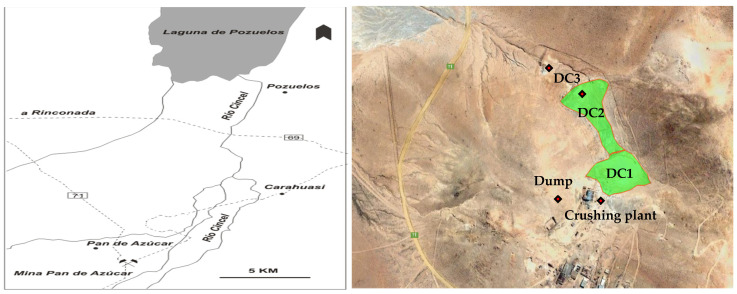
Location of Pan de Azúcar mine and Laguna Pozuelos (**left**); magnified view of the tailings dams in the area (**right**). The sampling points the red (♦) are indicated in the last view.

Pan de Azúcar mine is located in the northwest of Argentina 3700 m above sea level (at 22°32–22°38 S and 66°01–66°08 W) (Figure 1). The mineralization is hosted in hydrothermal veins intruded in dacitic rocks and dacitic breccias. The mineral in the mine presented high sulfide content including sphalerite and galena (with silver as inclusions) accompanied by abundant pyrite and marcasite. Quartz, illite, kaolinite, adularia, and scarce calcite are the hydrothermal minerals that hosted the ore. As was indicated above, from the three tailings dams with mining waste, only DC2 seems to be more exposed to the surface runoff mainly in the rainy season [25] and, that is why it was selected to analyze the behavior of the tailings dams in the area.

### 2.2. Acid Drainage Samples

In sterile flasks, acid mine drainage samples were collected in different locations of the mining area: tailings dam (DC2), crushing plant, lagoon located at the end of the tailings and dump (see Figure 1). As control, a downstream sample was also obtained from Cincel River that, as aforementioned, connects Pan de Azúcar mine with Laguna Pozuelos.

#### 2.2.1. Physicochemical Analysis

The pH, redox potential (Eh), and the concentration of the main ions in the acid drainage samples were measured. The two first parameters were determined in situ with multiparametric Hanna equipment. After filtering the samples were diluted using ultrapure HNO_3_ and analyzed by atomic absorption spectroscopy (Shimadzu AA6650F atomic absorption spectrophotometer) for cations and by ion chromatography (IC) for anions.

#### 2.2.2. Ecotoxicology Bioassays

The toxicity of the acid drainage samples was evaluated using standardized ecotoxicology bioassays (in triplicate) with *L. sativa* seeds according to specific methods (IRAM-29114:2008 and IRAM-259117:2009). The drainage with the lowest pH and the highest concentrations of metal ions (see below) was selected. The toxicity experiments were done by diluting AMD-1 using water from Cincel River (filtered through 0.22 μm pore size membrane); seeds were germinated at 20 °C. Germination was compared with a control in reconstituted water at 120 h [28]. In the case of positive germination, the seedlings were immediately removed and their length was measured and compared with that obtained in other control with water from Cincel River but without any contaminant, and the results were used in a dose-response analysis. Zinc sulfate was used as reference contaminant in toxicity control experiments. 

### 2.3. Tailings Samples

**Figure 2:**
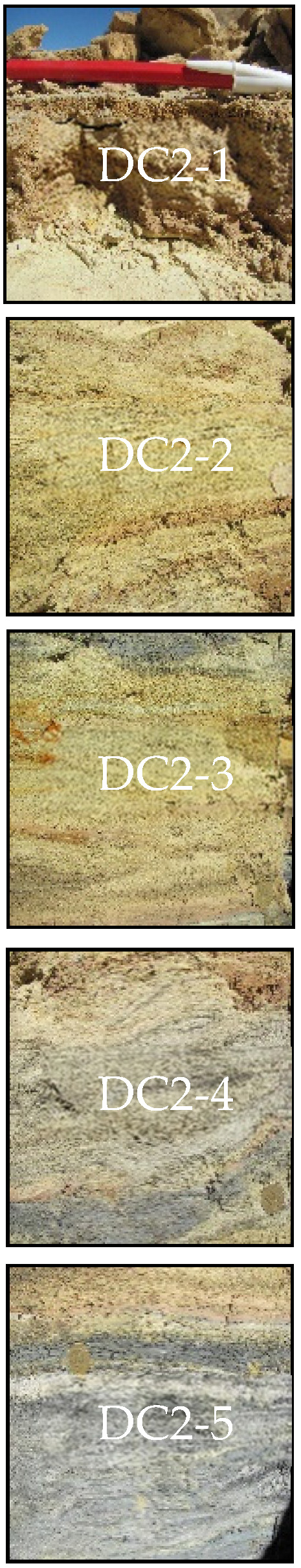
Horizontal profiles in DC2 dam.

Mineral samples were taken from DC2; for this purpose, pits of approximately 1 m^2^ were made until reaching the basement contact (about 1–1.8 m depth). Overall, five different horizons were detected mainly based on the color of the sediment in the profile and were sampled and numbered from 1 to 5 from the surface to the maximum depth reached (Figure 2). DC2-1 is an alluvial material that covers the tailings composed of fine sand with light brown gravel containing abundant sulfides. This layer is not the original tailing but the product of the erosion of material from the surrounding area and from the surface of the dam. DC2-2 sediments are reddish-brown clays, while DC2-3 sediments are fine yellowish sands intercalated with thin layers of whitish clays. DC2-4 and DC2-5 present grey sand and clays (darker in the second sample) with abundant sulfides.

Samples from each horizon were dried at room temperature after their mineralogical characteristics, color, and grain size were determined. The dry samples were homogenized and packed into polyethylene bags for storage at room temperature in a dry room. The pH, real and apparent density, porosity, and concentration of carbonates were determined in the lab for each horizon from the profile [29]. The pH value was determined by means of a specific electrode after contacting the sediment and water (1:2.5) for 1 h. Apparent density is the mass per unit apparent volume and real density is the mass per unit volume excluding that corresponding to closed and open pores. Porosity was calculated from the ratio between apparent and real densities. Carbonate determination was carried out according to ISO 10693:1995.

### 2.4. Prediction of AMD Generation

#### 2.4.1. Physicochemical Tests

The NAG test (Environmental Geochemistry International, EGI) was performed on the samples. For this purpose, 1 g of each sample was placed in a 250 mL Erlenmeyer flask containing 100 mL of H_2_O_2_ 15% *v*/*v* as an oxidizing agent. After leaving the mixture overnight, it was kept boiling for 2 h with permanent addition of water to maintain the same volume. NAG pH is the pH determined in the final solution. This solution was titrated with NaOH to determine the NAG value [30].

#### 2.4.2. Biooxidation Tests Using Collection Strains

As indicated, microorganisms have a fundamental role in the formation of acid drainage; for that reason, the biooxidation of the samples using microorganisms with bioleaching capacity could constitute an alternative to the classic methods to predict the risk of acid drainage formation [31]. Biooxidation tests were performed on each of the samples from DC2 (DC2-1, DC2-2, DC2-3, DC2-4, and DC2-5). The tests were carried out in 250 mL Erlenmeyer flasks with a final volume of 150 mL of minimum medium called 0K (KCl 0.1 g·L^−1^; MgSO_4_·7H_2_O 0.5 g·L^−1^; K_2_HPO_4_ 0.5 g·L^−1^; Ca(NO_3_)_2_ 0.01 g·L^−1^; (NH_4_)_2_SO_4_ 3 g·L^−1^) using two different pulp densities (2 and 5 % *w*/*v*). Flasks were inoculated using a combination of pure cultures of the collection strains, *Acidithiobacillus ferrooxidans* DSM 11477, *Leptospirillum ferrooxidans* ATCC 29047, and *Acidithiobacillus thiooxidans* DSM 11478. Previously, the first two species were grown in 9 K medium (pH 1.8) while the last one was grown in 0 K medium (pH 2.0) in shaken and thermostatized flasks (150 rpm, 30 °C). Once the exponential growth phase was reached, the cultures were centrifuged and washed 3 times with acidified distilled water to eliminate traces of the previous medium. The microorganisms were then resuspended in a certain volume of 0 K medium and finally mixed in equal parts (with a similar number of cells for each species).

Uninoculated controls were also prepared by replacing the volume of inoculum with fresh medium. All vials were incubated in a thermostatically controlled room at 30 °C and shaken at 150 rpm. During the test, samples were taken at different times. 

The o-phenanthroline method was used to determine Fe(II) concentration. To determine the total soluble Fe concentration, the samples were diluted with 0.14 M HNO_3_ and filtered with 0.45 µm pore diameter membranes to remove possible suspended solids, and then measured by atomic absorption spectroscopy (Shimadzu AA6650F atomic absorption spectrophotometer).

### 2.5. Enrichment, Isolation, and Microbial Characterization

All samples collected in the area (acid drainages, sediments, and samples from the tailings) were enriched in media with iron(II) or sulfur, obtaining positive results of microbiological growth. Samples taken from sediments, tailings, and drainages were enriched to detect specific microbial activity. For that purpose, 1 g of each solid sample or 5 mL of liquid samples was added to 100 mL of 0 K medium with the addition of 10 g·L^−1^ of elemental sulfur or 9 g.L^−1^ of iron(II) (as iron(II) sulfate), in both cases at an initial pH equal to 2.0. The vials were incubated at 150 rpm and 30 °C. The eventual positive growth was monitored with periodic measurements of pH, Fe(II) concentration by permanganometry and optical microscope observations. Once growth was observed, cultures were replicated at 10% in the corresponding fresh medium. In the exponential growth phase, 5 mL of the culture were concentrated by centrifugation at 7000 rpm to be used in the DNA extraction procedure. The pellet obtained was washed twice with distilled water at pH 2 and a third time with TE buffer, in order to remove traces of heavy metals that could interfere in the subsequent processes.

The Fast DNA Spin kit for soil (Bio 101, Carlsbad, CA, USA) was used for DNA extraction of the cultures. The genomic DNA was eluted in 70 μL high purity distilled water and stored and preserved at −20 °C until its later use. Polymerase chain reaction (PCR) amplifications of the 16S rRNA genes were performed using the bacterial-specific primers (341F-GC; 907). Amplifications were confirmed by an electrophoretic run (1.2% agarose gel; intensity, 90 V·cm^−1^; TAE buffer 1X; time, 30 min) and ethidium bromide stains (1 μg·mL^−1^) were performed for DNA fragment visualization (UV light transillumination from λ equal to 260 nm) [32]. On the other hand, amplifications performed to detect archaea were not successful [33].

In order to analyze the biodiversity, the 16S rRNA genes amplified were separated using the Denaturing Gradient Gel Electrophoresis (DGGE) technique; 12 µL of the PCR products were loaded in a polyacrylamide gel ammonium persulfate solution (APS) with a concentration of 0.1 g·mL^−1^ and N,N,N’,N’ Tetramethylethylenediamine (TEMED) were used as polymerizing agents and urea and formamide as denaturing agents in a 40–70% gradient). Electrophoresis was performed using CBS Scientific DGGE-2401 in a cube at a potential of 100 V for 16 h. After the run the gel was stained using SYBR Green and visualized by a Bio-Rad UV transilluminator; the digital images of the gels were captured with the Quantity One software (Bio-Rad Laboratories, Inc., Hercules, CA, USA). The main bands were cut from the polyacrylamide gel using a sterile scalpel and transferred to sterile Eppendorf tubes containing 100 μL of sterile water. The DNA was eluted as described in [34] and re-amplified using the 341F-907R primer set. The PCR products and integrity were checked by gel electrophoresis and ethidium bromide stain and were finally sent to Macrogen Inc. (Seoul, South Korea) for sequencing. The sequences were analyzed and compared with NCBI database using BLAST software [31]. Based on these results a new DGGE was run (using exactly the same procedures described above) adding amplification products of the 16S rRNA genes obtained from cultures of collection strains (see Section 2.4.2) of *A. ferrooxidans*, *A. thiooxidans,* and *L. ferrooxidans* to compare its positions with those of the bands of the samples.

To confirm the preliminary identification made from the DGGE bands, the DNA of the samples was amplified using the 27F-1542R primer set for the almost complete 16S rRNA gene. The PCR products that showed satisfactory purity and integrity in an electrophoretic run stained with ethidium bromide (as described above) were cloned using the TOPO TA Cloning Kit (Invitrogen, CA, USA) following the manufacturer’s instructions. Based on the low number of bands observed in the DGGE profile that indicates low bacterial diversity, only a few clones were chosen from each sample (see complete procedure in [35]). Sequencing of the 16S rRNA genes was performed by the Macrogen service (Macrogen Inc., Seoul, South Korea).

For a preliminary quantification in the sulfur- and iron-oxidizing consortia, FISH was applied using specific probes for the bioleaching microorganisms (*A. thiooxidans*, *A. ferrooxidans*, *L. ferrooxidans*) detected previously; 5′ indocarbocyanine dye (CY3) was used as fluorophore. Hybridization with the probes was carried out on membranes where the samples from the different consortia were fixed [33].

### 2.6. Biooxidation Tests Using Native Consortia

Sulfur- and iron-oxidizing enrichment consortia obtained from environmental samples or a mixture of both, were used to perform another series of biooxidation tests for samples from DC2. The preparation of the inocula and the conditions used in the experiments were similar to those described for the pure cultures (see Section 2.4.2).

## 3. Results and Discussion

Table 1 shows the pH value and the concentrations of the main ions present in two of the acid drainages collected in the area (AMD-1 corresponds to the sample from the crushing plant, while AMD-2 was taken from the lagoon associated with the tailings). The values determined for the other acid drainages were between those corresponding to AMD1 and AMD2 (data not shown). The last columns of the table show the data for Cincel River and the guideline values by U.S. Environmental Protection Agency (EPA).

As expected, the acid drainages, and in particular the one sampled at the crushing plant, showed extremely low pH values and very high concentrations of iron, zinc, and sulfate. Moreover, concentrations were well above the limits allowed by U.S. EPA for cadmium and lead (also for nickel in some cases) which are very toxic metals. On the other hand, the concentrations determined for Cincel River were below the permitted values (or, at least, in some cases, such as Cd and Pb, below the detection limits of the technique used in this study), indicating it was not contaminated at least in dry season when the campaign was done. However, in the rainy season, rainwater which runs off through the tailings and the area where the acidic drainages were detected, can reach the river and possibly the entrance of AMD with high acidity and high concentrations of toxic metals could modify the parameters of the river (not confirmed because sampling on the same points during the rainy season was not possible).

In order to determine the possible ecotoxicological influence that the acid drainage discharge could have on Cincel River, the bioassay with seeds of *L. sativa* L for determining acute toxicity was performed. This test uses lettuce because of its high sensitivity to many toxic species, rapid and uniform germination, and it is a cheap, simple, very versatile method that has proven to be representative of the effect of different contaminants on biological systems and, in particular, on seeds and plants [37]. In this case, the seeds were exposed to AMD-1 acid drainage by progressively diluting it with the water from Cincel River. After 120 h, the relative germination percentage was determined comparing with a control in reconstituted water with no contaminant added. In times subsequent to the one considered, additional germinations were observed in certain dilutions of the acid drainage, although without reaching the germination level of the control. Thus, in the analysis of the bioassay, the relative stem height of the seedlings after 120 h was used (referred to the control with water from Cincel River without contaminant). From these values, the half maximal inhibitory concentration (IC_50_) was determined, i.e., the concentration needed to inhibit a biological process (in this case, the development of the seedling) by 50%.

**Figure 3:**
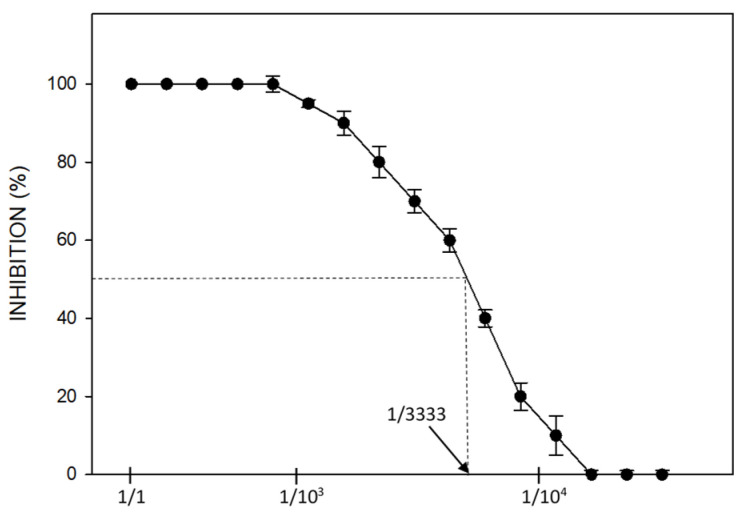
Effect of AMD-1 at different dilutions on seedling development. Dilution ratio is indicated as 1/X where X is the final volume after dilution.

Figure 3 shows the ecotoxicological effect of increasing dilutions of AMD-1. It can be seen that only dilutions higher than 1 in 1000 allowed partial germination and seedling development. The IC_50_ value obtained from the graph indicates that even dilutions higher than 1 in 3000 produce enormous ecological damage. Finally, dilutions higher than 1 in 10,000 (and up to 1 in 100,000 which was the maximum dilution used) did not provoke statistically significant modifications in the seedling development compared with the control with water from Cincel River without AMD addition.

**Figure 4:**
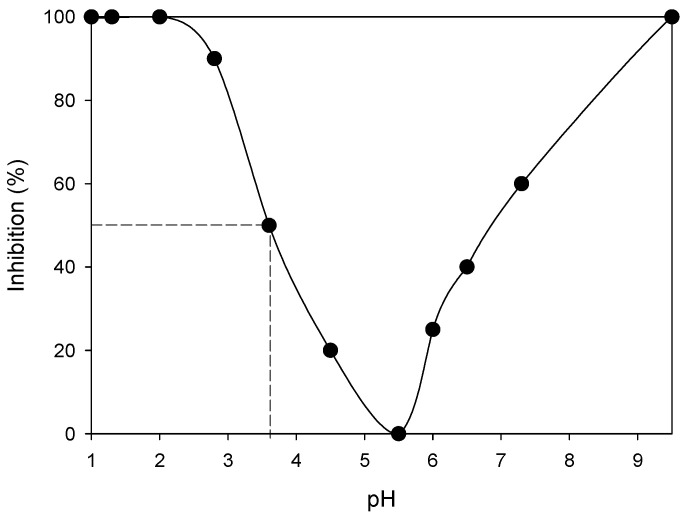
Effect of pH on seedling development.

A dilution of almost 4 orders of magnitude, as necessary to reach IC_50_, significantly reduces the concentrations of iron and zinc, but they are still at levels much higher than those recommended by U.S. EPA; those species do not present a serious health risk although such concentrations are unacceptable. On the other hand, with the indicated dilution the level of lead is below the maximum limit for water for human consumption. The cadmium present in AMD, even after such dilution, still has a concentration higher than the maximum admitted and could therefore be responsible for the ecotoxicological effect observed. In addition, the acidity of AMD could also be a source of toxicity. In order to see the effect of pH, samples of water from Cincel River were adjusted with sulfuric acid solution (or potassium hydroxide solution) up to the required pH. The toxicity experiments were carried out in similar way to those described to analyze the effect of AMD-1 on Cincel River. Figure 4 shows the results. It can be seen that the minimum inhibition occurred at approximately 5.2, which is close to the optimum pH for lettuce growth, and that 50% inhibition (IC_50_) occurred at a pH value of approximately 3.6, which is very close to that determined (3.64) when AMD is diluted to the level indicated by IC_50_. There also was a partial inhibition of seedling development at pH values higher than the optimum. However, since Figure 3 shows, dilutions higher than 1 in 10,000 (that decreases the pH of the water from Cincel River to values close to the optimum) did not produce neither inhibition nor improvement of the seedling development. These results indicated that the acidity produced for the addition to the water from Cincel River, although partially responsible, is not enough to justify the ecotoxicological effect; this should be related to the presence of cadmium and, to a lesser extent, lead and other metals and chemical species present into the AMD.

Table 2 lists the main characteristics of the five types of samples obtained from the profiles of DC2. Except for the first layer, which is significantly less porous, the soil is sufficiently porous for percolation to allow water to access the lower layers. In the extraction of the samples, once the depth of 100 cm was exceeded, it was found that a gradual increase in humidity turned the material muddy at depths close to 160 cm. Chemical and mineralogical analysis on similar samples from the tailings were performed by Murray et al. [27]; the upper layers belong to the oxidation zone and mainly contained quartz, albite, illite, jarosite, schwertmannite, and gypsum, while the deeper layers contained pyrite, marcasite, sphalerite, galene, arsenopyrite, illite, quartz, and albite, among others. They also carried out a sequential extraction procedure on the samples determining the contents of the main metals: 2.57–16.24 wt% Fe, 2605–11,013 mg·kg^−1^ Pb, 310–1356 mg·kg^−1^ Zn, 211–1211 mg·kg^−1^ As, 2–9 mg·kg^−1^ Cu, 4–43 mg·kg^−1^ Cd, 20.3–1030 mg·kg^−1^ Cr. In addition, the sulfur content was in the range between 2.51 and 13.1 wt% S.

Table 3 shows the results obtained from the NAG test performed. NAG pH has been proposed as single indicator (or in combination with other static tests) of the potential of the samples to generate acidity; samples with NAG pH below 4.5 are potentially acid forming (PAF) [38,39,40]. This parameter is generally more appropriate since it is the result of the balance between the ability to produce acid and the neutralizing capacity of the rocks, while other parameters calculate the potential to generate acid from the sulfur content by overestimating that capacity since there are sulfur-containing minerals (including many sulfides) that are not acid-producing minerals.

Although all samples were characterized as potentially acid forming (PAF), the deeper fractions were those with higher production capacity. Based on the NAG values (i.e., the acid production calculated from the titration of the solution up to a pH 4.5 value) the samples were classified as low or high capacity acid-forming material. Although the measurement of NAG pH is simpler, in these samples the NAG value seems to be more representative of the environmental risk of generating acid drainage.

DC2-1 showed a markedly different behavior giving higher values to its two subsequent layers. This is consistent with the fact that this layer originated as a product of the erosion of the material from the surrounding areas after the mine closure and it is composed of a mixture of primary and secondary minerals and alluvial sediments (about 29% of Fe was extracted in oxy-hydroxides and oxides fractions, while 65% was extracted using an oxidizing action).

DC2-2 and DC2-3 were classified as samples with low capacity to generate acid. Although both had low NAG pH values, they did not show great capacity for acid generation. These results are consistent with those of Murray et al. [27] since samples DC2-2 and DC2-3 correspond to an essentially oxidized zone where iron was mainly extracted (83 and 86%) in the iron(III) oxyhydroxides and oxides fractions (extracted using ammonium oxalate at room temperature and at 80 °C, respectively); sulfur was extracted in the water-soluble fraction (sulfates, probably) and also together with Fe in the oxyhydroxides and oxides fractions, which would indicate its participation in phases such as jarosite. The absence or, at least, the low content of pyrite and marcasite explains why these sediments have a low capacity to generate acidic drainages in agreement with the NAG analysis.

On the other hand, DC2-5 belongs to the primary zone where almost 90% of Fe and S (together with other elements) are present in the sulfide phase since the highest percentage was extracted in the oxidizing attack (performed using H_2_O_2_ to dissolve secondary sulfides and a mixture of KClO_3_ and HCl and subsequently 4 M HNO_3_ at boiling to dissolve primary sulfides). Evidently these sediments with a high pyrite and marcasite content have a potential for the generation of acid drainages. 

Finally, DC2-4 is a transition zone between the oxidized and the primary zones. The acid production of this sample was surprisingly high considering that its NAG pH was quite high, which probably indicates an important buffer effect due to its composition.

**Figure 5:**
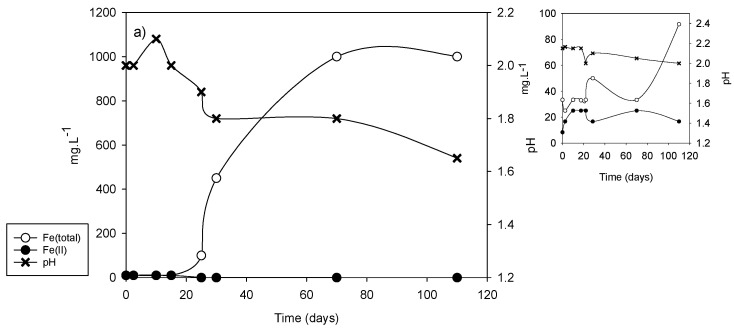

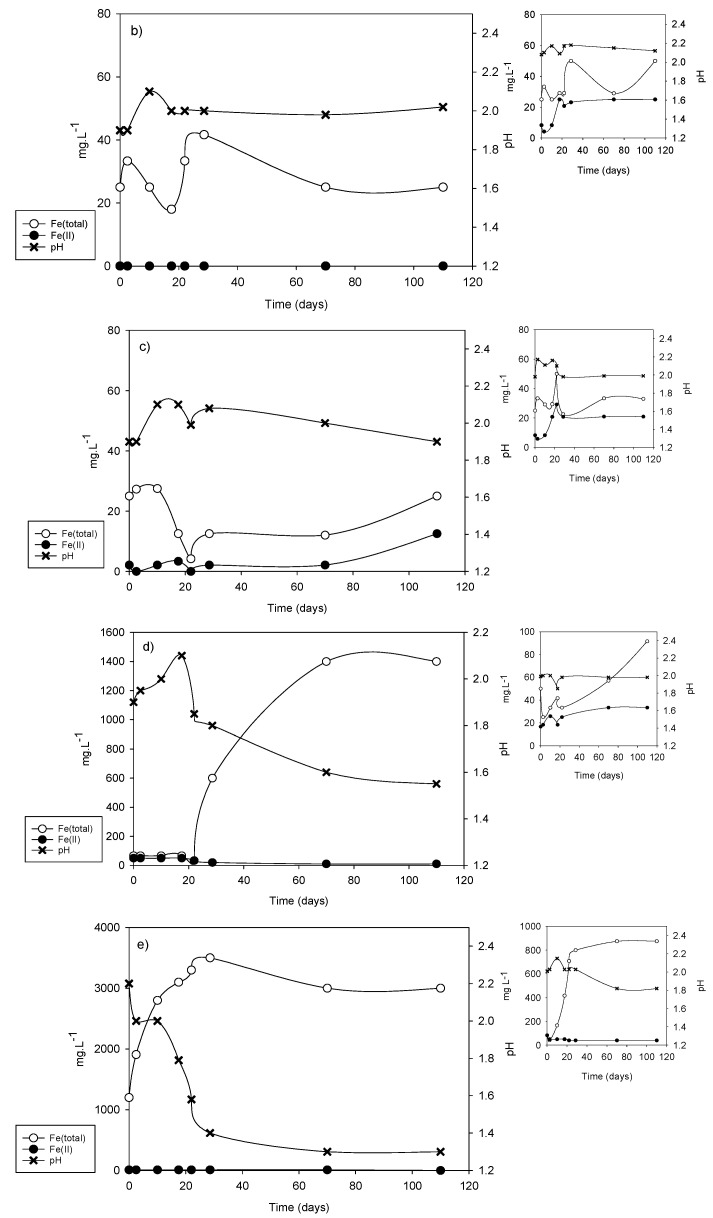
Biooxidation of samples from the horizontal profiles in DC2 dam (pulp density: 2% *w/v*). (**a**) DC2-1; (**b**) DC2-2; (**c**) DC2-3; (**d**) DC2-4; (**e**) DC2-5. Evolution of total soluble iron and ferrous iron concentration, and pH in inoculated **(left**) and uninoculated (**right**) systems.

As indicated, microorganisms have a fundamental role in the formation of acid drainage; for that reason, an alternative to the classic methods to predict the risk of acid drainage formation is the biooxidation of the samples using microorganisms with bioleaching capacity [31]. Figure 5 shows the results of the tests carried out with an artificial consortium of microorganisms using collection strains (*A. ferrooxidans*, *A. thiooxidans,* and *L. ferrooxidans*); in the smaller graphs the behaviors of the corresponding uninoculated systems are shown.

As can be observed in Figure 5, DC2-5 and to a lesser extent DC2-1 and DC2-4 were significantly biooxidized, causing an increase in the total iron concentration (exclusively as Fe(II)) and a pronounced decrease in the pH, indicating a strong microbiological oxidizing action.

In uninoculated systems, the maximum soluble iron concentration was achieved after 70 days, solubilization was barely relevant for DC2-5 (although much lower than that corresponding to the inoculated system) after 15 days; for DC2-1 and DC2-4 an incipient solubilization was observed after 70 days. Before the increases in the solubilization, iron in solution was essentially ferrous but later, the concentration of ferric ion increased. This strongly suggests that this solubilizing action was produced by the action of native microorganisms since the sediments were not sterilized.

From Figure 5, two parameters were collected in order to compare with NAG to predict the generation of acid mine drainage: rate of microbial-promoted iron solubilization and pH in the solution after the time considered. In our case, we tested three different times (30, 70, and 110 days) to decide which the most convenient was in order to select the shortest possible (see Table 4).

**Figure 6:**
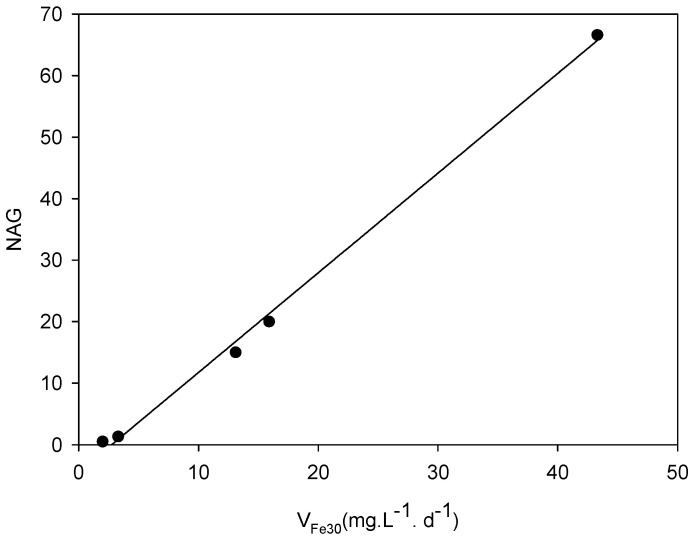
Biooxidation rate vs. NAG pH of the samples.

The results in Table 4 show that although the absolute values and also the relative values can change if different biooxidation times are used, the differences between the different samples remain. The samples for which the biooxidation process lowers the pH are potential generators of acid drainage; for those samples, the risk of acid drainage increases directly with the value of the average iron solubilization rate. Figure 6 shows the latter parameter and the NAG value for each sample. A very good correlation between both parameters is observed (r^2^ = 0.99). There is also a good correlation with the sulfide net neutralization potential (SNNP) calculated by Murray et al. [27]; SNNP values for DC2-2 and DC2-3 were just below zero, while for the other samples the values were negative.

**Figure 7:**
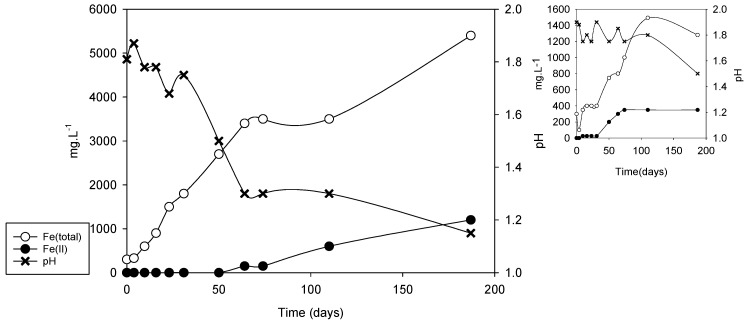
Biooxidation of DC2-5 sample (pulp density: 5% *w*/*v*). Evolution of total soluble iron and ferrous iron concentration, and pH in inoculated (**left**) and uninoculated (**right**) systems.

The change in slurry density used in the experiment was analyzed with the DC2-5 sample, which is the one with the highest risk of generating acid drainage within the profiles used. Figure 7 depicts the corresponding curves for a density of 5% *w/v*. A behavior very similar to that corresponding to the lower pulp density can be observed except that a greater solubilization is reached and the time required for this is greater than that for 2% *w/v* (it did not even reach a clear plateau after 150 days). In addition, lower pH values are reached, less than 1.3, which are manifested as an increase in the concentration of ferrous iron since its oxidation catalyzed by certain iron-oxidizing microorganisms such as *A. ferrooxidans* is inhibited in such acidic media [41]. The average rate of iron solubilization reaches a value of about 50 mg·L^−1^·d^−1^ at 30 days, which is quite close to the value reached with pulp density of 2% *w*/*v*, suggesting that pulp density in the biooxidation experiments does not limit the use of the rate of iron solubilization to predict the risk of generating acid drainage.

It is interesting to note that also here, the solubilization in the uninoculated system begins to increase significantly between 30 and 40 days of the test. A significant iron solubilization value is reached after approximately 100 days and then, a slight decrease coincides with the decrease in pH that may be linked to the precipitation of solid iron(III) phases.

Samples collected in the area (including acidic drainages, sediments, and samples from the tailings) were enriched in media with iron(II) or sulfur, obtaining positive results of microbiological growth. The enrichments were repeated several times in the corresponding medium to obtain sulfur- and iron-oxidizing consortia. A low number of bands in the DGGE gel (data not shown) indicated low biodiversity in the samples; sequences from the most intense and repetitive bands in the different of the consortia corresponded to *A. ferrooxidans*, *A. thiooxidans*, and *L. ferrooxidans*. That was later confirmed by running a new DGGE comparing the position of the 16S rRNA gene bands of collection strains of such species and the samples and by the results of the sequencing of the 16S rRNA gene clones obtained from the different consortia.

On the other hand, a 100% hybridization with the EUB388 probe (data not shown) was obtained for all the consortia, indicating that the microorganisms correspond to the bacterial domain. In all sulfur-oxidizing consortia, positive FISH hybridization was obtained with specific probes for *A. thiooxidans* while just in some consortia *A. ferrooxidans* was also detected although with hybridization percentages of 10% or less (data not shown). In the iron-oxidizing consortia, positive FISH hybridization was obtained mainly with the specific probe for *A. ferrooxidans* and only in a couple of cases, and to a lesser extent (hybridization percentage lower than 15–20%), for *L. ferrooxidans* (data not shown).

The three species detected (*A. ferrooxidans*, *A. thiooxidans*, and *L. ferrooxidans*) have frequently been isolated from acidic drainages and, furthermore, have been shown to be often dominant in the processes of their generation [7,10]. Although the consortia were classified according to the energy source that was used in the enrichment, they may contain species with the ability to oxidize other energy sources including that used in the other enrichments. One of these possible species is, obviously, *A. ferrooxidans* due to this iron- and sulfur-oxidizing bacteria was detected in most of the iron-oxidizing consortia and also in some sulfur-oxidizing consortia.

The two sulfur-oxidizing native consortia (those that showed the fastest kinetics to oxidize sulfur in vials) and the other two iron-oxidizing native consortia (those capable of oxidizing iron(II) more quickly) were used, individually or in combination, in biooxidation experiments of DC2-5 which was classified as the sample with the highest risk of generating acid drainage.

**Figure 8:**
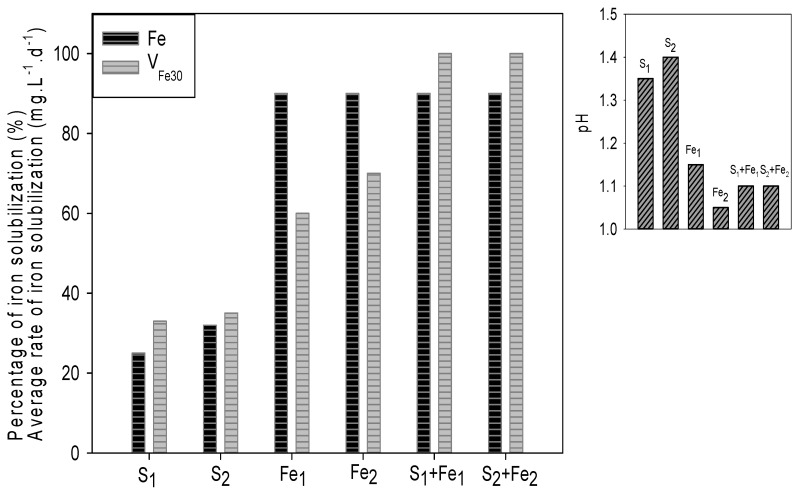
Biooxidation of DC2-5 sample using native consortia (pulp density: 5 % *w/v*). Rate of iron solubilization and percentage of iron solubilized (**left**) and final pH (**right**) after 120 days.

The behavior of biooxidations was qualitatively similar to that obtained with the collection strains. Figure 8 shows the final pH and percentage of Fe solubilization reached at the end of the experiment (120 days) and the average iron oxidation rates obtained in the first 30 days.

The comparison between the different systems indicates that the solubilization of iron requires the participation of an iron-oxidizing consortium (although it also contains sulfur-oxidizing species); when the consortium only contains sulfur-oxidizing microorganisms (or at least when the iron-oxidizing species do not dominate the consortium), the extraction is significantly lower (around 30% of that achieved by the other consortia). This is because, as mentioned, solubilization of the iron present in pyrite and marcasite requires an oxidizing microbial attack. The extraction of iron in the systems inoculated with the sulfur-oxidizing consortia increases after 60–80 days of biooxidation, which could indicate the activity of native iron-oxidizing microorganisms present in the sediments (which had not been sterilized).

On the other hand, the systems inoculated with the iron-oxidizing consortia or with mixed consortia, achieved a very high extraction of iron (close to 90% of the total present in the sediments) and much higher (above 50%) than that achieved by the consortia built with collection strains, which is probably linked to the tolerance to toxic species present in the sediments. Although the final extraction was similar in the cultures with iron-oxidizing consortia and with mixed consortia, the extraction kinetics was slightly different. The mixed consortia were faster in the first 30–40 days but then the rate of solubilization decreased; those inoculated exclusively with iron-oxidizing consortia reached maximum solubilization at 80–100 days while the mixed ones only did so between 100 and 120 days. The significant increase in solubilization that was observed in the mixed cultures in the first 20–30 days seems to be related to a probable higher activity of the sulfur-oxidizing microorganisms than that in iron-oxidizing consortia, provoking a faster reduction in pH and a greater initial dissolution. However, the increase in ferric ion concentration caused an abundant precipitation of solid phases of iron that partially covered the surface of the minerals, reducing the rate of the later attack. Although the final extraction was different, the average rate in the first 30 days was equal (in the iron-oxidizing consortia) or higher (in the mixed consortia) than the one reached with the consortium built with collection strains.

The biooxidation methodology used in this work is slightly different from the one proposed by Bruynesteyn [31] and described explicitly by the Environmental Protection Agency Technical Report (as British Columbia Research Confirmation Test) since the inoculum used contained three microbial species (while the original method proposed inoculation with *A. ferrooxidans* only). Our results show that biooxidation studies allow us to obtain results comparable to those achieved with other methodologies to predict acid drainage. In any case, this methodology is scarcely used to evaluate the risk of generating acid drainage and in many reviews it is not even included as a possible methodology, when in fact microorganisms have a preponderant and decisive role in the generation of acid drainage [30,42,43,44].

One of the criticisms of this methodology is that it requires much longer time than the static tests and that an initial acidification is performed, which is important to allow the activity of acidophiles. Actually, this methodology is closer to kinetic tests since it mimics natural conditions or even more because it includes irreplaceable protagonists such as microorganisms. On the other hand, the times required are not very compatible with the need for quick and simple tests, but they can be considerably reduced by increasing the size of the inoculum, reducing the density of the pulp, and using more suitable conditions for microbial development. Furthermore, using native populations rather than collection strains makes the method much more interesting; native populations will surely show faster biooxidation kinetics (as observed in our results), tolerance to eventual toxic species that could inhibit the collection strains and, if enrichments are used and not isolated (as in this work), possible contributions of other microbial species that could be present are not discarded or minimized. In addition, biooxidations with native microbial populations are an accelerated version (by adaptation of the conditions) of the processes of acid drainage generation that can occur in the field.

In this work it has been demonstrated that the superficial zone of Pan de Azúcar tailings (5.5 wt% of the total weight of residues in DC2 dam) and the deeper zones that include the primary zone (75.5 wt% of the total weight of residues in DC2 dam) of such tailings have a high capacity to generate acid. This was confirmed by NAG assays and also by the results of biooxidations performed with collection strains of leaching microorganisms. Native leaching microorganisms are ubiquitous in the area and the consortia obtained from the enrichment of samples showed even greater biooxidation capacity than the collection microorganisms, strongly suggesting that the risk of generating acid drainage in the dam is very high. The acidic drainages generated at the site showed a high ecotoxicological effect and constitutes a risk for the nearby Ramsar site (Laguna de Pozuelos) that is hydrologically connected to Pan de Azúcar mine.

## Figures and Tables

**Table 1:** Composition of acid mine drainages (N.D.: not determined; N.P.: not proposed).

	AMD-1	AMD-2	Cincel River	Guideline Values (US EPA) [36]
**pH**	0.58	2.25	8.16	6.5–9.5
**Fe (g·L^−1^)**	97.40	0.860	<3.10^−5^	2.10^−4^
**Zn (g·L^−1^)**	25.10	0.214	<5.10^−6^	5.10^−3^
**Pb (mg·L^−1^)**	1.30	<0.2	<5.10^−2^	1.10^−2^
**Cd (mg·L^−1^)**	161	1.4	<2.10^−2^	5.10^−3^
**Ca (mg·L^−1^)**	<2	9.2	11.4	N.P.
**Ni (mg·L^−1^)**	5	0	<5.10^−2^	2.10^−2^
**Mg (mg·L^−1^)**	320	23.2	4.4	N.P.
**Na (mg·L^−1^)**	517	2.0	25.7	20
**K (mg·L^−1^)**	<1	1.3	0.9	N.P.
**SO_4_^−2^ (g·L^−1^)**	42.30	3.98	N.D.	0.250

**Table 2:** General characteristics for the tailings samples.

	DC2-1	DC2-2	DC2-3	DC2-4	DC2-5
**Depth (cm)**	0–14	14–26	26–50	50–66	>66
**Estimated area (m^3^)**	3668	3144	6288	4192	35111
**Estimated mass (tons)**	7.6	8.6	17.6	12.1	92.0
**pH**	2.69	2.43	2.00	2.12	1.90
**Apparent density (g·cm^−3^)**	1.53	1.09	1.24	1.44	1.58
**Real density (g·cm^−3^)**	2.07	2.73	2.80	2.88	2.62
**Porosity**	26.1	60.1	55.7	50.0	39.7

**Table 3:** NAG classification of the samples from DC2 profile.

Sample	NAG_pH_	NAG *	Type
**DC2-1**	2.01	13.1	Potentially acid forming (PAF)
**DC2-2**	2.62	3.3	Potentially acid forming-low capacity (PAF-LC)
**DC2-3**	2.66	2.0	Potentially acid forming-low capacity (PAF-LC)
**DC2-4**	3.90	15.9	Potentially acid forming (PAF)
**DC2-5**	1.74	43.3	Potentially acid forming (PAF)

* kilogram of sulfuric acid per ton of sample.

**Table 4:** Main parameters of the biooxidation of DC2 samples.

Sample	v_Fe30_ (mg·L^−1^·d^−1^)	pH_30_	v_Fe70_ (mg·L^−1^·d^−1^)	pH_70_	v_Fe110_ (mg·L^−1^·d^−1^)	pH_110_
**DC2-1**	15.0	1.80	14.28	1.65	9.1	1.60
**DC2-2**	1.33	2.05	0.35	2.05	0.22	2.10
**DC2-3**	0.5	2.00	0.34	2.00	0.22	2.00
**DC2-4**	20.0	1.75	20.0	1.60	12.72	1.60
**DC2-5**	66.6	1.50	27.87	1.30	17.72	1.40

v_Fe”t”_: average rate of iron solubilization at 30, 70, or 110 days; pH “t”: pH after 30, 70, or 110 days.

## Data Availability

Not applicable.
